# Neutrophilic inflammatory enteropathy in 27 dogs: a retrospective descriptive study

**DOI:** 10.1093/jvimsj/aalag130

**Published:** 2026-07-08

**Authors:** Kevin Murtagh, Pamela Kelly, Robert Shiel, Emma J O'Neill, Carmel Mooney

**Affiliations:** Department of Veterinary Small Animal Clinical Studies, University College Dublin, Dublin, Ireland; Department of Veterinary Pathobiology, University College Dublin, Dublin, Ireland; School of Veterinary Medicine, Murdoch University, Murdoch, Australia; Department of Veterinary Small Animal Clinical Studies, University College Dublin, Dublin, Ireland; Department of Veterinary Small Animal Clinical Studies, University College Dublin, Dublin, Ireland

**Keywords:** chronic inflammatory enteropathy, hypocobalaminemia, melena, neutrophilic inflammatory enteropathy, peripheral neutrophilia

## Abstract

**Background:**

Dogs with chronic inflammatory enteropathy (CIE) are common in companion animal practice. Neutrophilic inflammatory enteropathy (NIE) is a subtype of CIE that is uncommonly reported, posing a dilemma in terms of etiology, treatment, and prognosis.

**Hypothesis/Objectives:**

To describe historical, clinical, clinicopathological, imaging findings, treatment, and survival in dogs with histologically confirmed NIE.

**Animals:**

Twenty-seven client-owned dogs with NIE.

**Materials and methods:**

Retrospective interrogation of the hospital database between January 2015 and January 2025 identified dogs with NIE based on histological reports. Cases were regraded using modified WSAVA guidelines and classified into the minor (mild inflammation) or major (moderate or severe inflammation) groups.

**Results:**

Twenty-seven dogs were identified, with 8 and 19 dogs in the minor and major groups, respectively. The mean age was 7.7 ± 3.5 (95% CI, 6.4-9.1) years. The most common presenting signs were diarrhea (*n* = 21/27; 78%), vomiting (*n* = 21/27; 78%), weight loss (*n* = 20/27; 74%), hyporexia/anorexia (*n* = 13/27; 48%), and melena (*n* = 7/27; 26%). Clinicopathological abnormalities included neutrophilia (*n* = 12/27, 44%), hypoalbuminemia (*n* = 14/27, 52%), hypoglobulinemia (*n* = 23/27, 85%), hypocholesterolemia (*n* = 11/27; 41%), total hypocalcemia (*n* = 11/27; 41%), and hypocobalaminemia (*n* = 14/24; 58%). Twenty-four (89%) dogs (6 minor and 18 major) survived to discharge, with an overall median survival time of 267 (95% CI, 0-569) days, with no statistically significant difference between the major and minor groups. Peripheral neutrophilia was associated with an increased hazard of death (4.43; 95% CI, 1.16-16.99; *P* = .03) on univariate analysis and a significantly shorter median survival time.

**Conclusion and clinical importance:**

Neutrophilic inflammatory enteropathy is potentially associated with poor survival. Peripheral neutrophilia might indicate a poorer prognosis.

## Introduction

Chronic inflammatory enteropathy (CIE) in dogs is characterized by persistent or recurrent signs of gastrointestinal (GI) disease lasting ≥ 3 weeks. The disease is considered to involve a complex interplay between host genetics, the microenvironment (microbiome and diet), and an aberrant immune response.^1^ Definitive diagnosis requires the exclusion of certain intestinal (obstructive and infectious) and extra intestinal (hepatobiliary, renal, endocrine, and pancreatic) diseases, while concurrently demonstrating histological evidence of intestinal mucosal inflammation.^2–4^ Despite the requirement to demonstrate intestinal inflammation to confirm a CIE diagnosis, biopsies of the GI tract are not always obtained. Rather, the histological diagnosis is presumed, and cases are classified clinically in terms of response to treatment as either food-responsive, microbiome-related modulation-responsive (previously called antibiotic-responsive), immunosuppressive-responsive, or, in some cases, nonresponsive enteropathies^5–7^; with food responsive enteropathy cases having the best long-term outcome.^8^ Antibiotic responsive enteropathy (ARE) was a term that described a cohort of dogs that had a favorable response to certain antimicrobials, such as tylosin,^9–11^ with reports suggesting a prevalence of 16%-33%.^2^^,^^5^ However, ARE is no longer an accepted term in veterinary gastroenterology because of concerns centered around antimicrobial stewardship, microbiome alterations, and transient responses in dogs treated with antimicrobial therapy. A large, recent retrospective study investigating chronic enteropathy identified no cases of ARE,^12^ supporting the premise that antibiotic therapy is not appropriate for dogs with chronic enteropathy.

While GI histology is often utilized for the diagnosis and to guide treatment of CIE in dogs,^13^ it is usually reserved for cases in which diet trials have not resolved the clinical signs or if there are negative prognostic factors present, such as hypoalbuminemia.^3^ The World Small Animal Veterinary Association (WSAVA) GI standardization group provided the first objective guidelines for biopsy assessment^4^^,^^14^; however, GI histology is often reported using a more simplified version of the original WSAVA guidelines.^13^ More recently, modifications to the original WSAVA guidelines have been published,^15^ which objectively classifies the inflammation as mild, moderate, or severe, reflecting the importance of individual histopathological variables, including the presence of neutrophils. It is clear from the current literature that the presence of neutrophils in the lamina propria is always abnormal^15^; however, it is not known whether their presence is part of the primary inflammatory disease or if they are recruited by other inflammatory cells or cytokines. Despite the wealth of information on canine CIE, there is a lack of information specifically regarding neutrophilic inflammation.

The aims of the present study were to describe a group of dogs diagnosed with neutrophilic inflammatory enteropathy (NIE) reporting on signalment, presenting clinical signs, clinicopathological data, imaging findings, treatments used before and after diagnosis, hospitalization time, and survival after diagnosis, based on the severity of neutrophilic inflammation identified.

## Materials and methods

### Inclusion criteria

Dogs with histologically diagnosed gastrointestinal neutrophilic inflammation, alongside lymphoplasmacytic inflammation, between January 2015 and January 2025, were identified by retrospectively searching a university referral teaching hospital pathology reporting database, using appropriate search terms. Cases with any neutrophilic inflammation were identified, with case records subsequently retrieved using 2 veterinary management software programs (Vetscope, version 2.27, Dublin, Ireland and ProVet, version 12.04, Nordhealth, Finland). Relevant results from each case were exported into a commercial spreadsheet software program (Microsoft Excel, Version 16.98) for analysis.

Each case had fresh histological sections cut from all available tissue and regraded by a single European-board-certified pathologist.^15^ Where certain tissue blocks were not available, regrading was performed, based on the original report. The mucosa was assessed for neutrophil, lymphocyte, and plasma cell number. The neutrophilic component within the gastric and small intestinal sections was graded as absent, mild, moderate, or severe (Appendix). An overall severity score of mild, moderate, or severe neutrophilic inflammation was assigned to each case based on the highest score from all available tissues. Each case was then allocated to a group of minor NIE, if the overall severity score was mild, and major NIE, if the overall severity score was moderate or severe. The lymphocyte and plasma cell populations within the same GI biopsies (Appendix) were graded and cases categorized similarly.^15^

All cases were presumed to have chronic enteropathy. Small intestinal diarrhea was appropriately defined (Appendix).

### Data collection

All cases had blood tests performed and recorded, using appropriate reference ranges (Appendix). Hypocobalaminemia was defined as serum cobalamin concentration < 275 ng/L, similar to the cut-off used by the Texas A&M University Gastrointestinal Laboratory.^16^ When a value < 150 ng/L (lowest limit of detection) and > 1000 ng/L (highest limit of detection) was identified for serum cobalamin concentration, a value of 150 and 1000 ng/L, respectively, was assigned. When a value > 24 μg/L (highest limit of detection) was identified for serum folate concentration, a value of 24 μg/L was assigned. When a cortisol value of < 1 μg/dL (lowest limit of detection) was identified, a value of 1 μg/dL was assigned.

Relevant abdominal ultrasound changes, treatments, and outcome measures were recorded (Appendix). Fluorescent in situ hybridization (FISH) analysis was performed at Cornell University, USA, using a standard eubacterial probe initially and if a positive fluorescence was identified, additional, more specific, bacterial probes were used.^17^

## Statistical analysis

Statistical analysis was performed using commercially available statistical software packages (SPSS, version 29 and GraphPad Prism, version 11). Continuous data, including age, weight, and clinicopathological results, were assessed for normality by visually comparing mean, median, and SD and, where appropriate, Shapiro–Wilk testing was performed. These data were expressed as mean ± SD and 95% CI or median (IQR), as appropriate. Comparison between groups was performed using the independent *t*-test or the Mann–Whitney *U* test. Univariate Cox regression analysis was performed to serve as a foundation for more complex multivariate analysis. Relevant variables, including the presence of peripheral neutrophilia, hypoalbuminemia, hypocholesterolemia, hypocobalaminemia, and total hypocalcemia, and for treatments used, including glucocorticoid and antibiotic therapy were assessed. Variables in which a *P* < .2 was identified were entered into a multivariable Cox regression model to determine overall significance as long as more than 1 variable existed. Kaplan–Meier survival curves were generated for significant variables identified on univariate analysis, and the log rank test was performed to assess differences between groups. A *P* value < .05 marked the threshold for an acceptable type 1 statistical error.

## Results

### Cases

A total of 27 cases with neutrophilic gastrointestinal inflammation were identified. Demographic data are detailed in Table 1.

**Table 1:** Demographic data for the 27 neutrophilic inflammatory enteropathy dogs included in this study. Sex is recorded as M, MN, F, and FN.

**Demographic factor**	**Result (*n* = 27)**
**Mean age in years ± SD (95% CI)**	7.7 ± 3.5 (6.4-9.1)
**Sex and neuter status**	M-8, MN-8
	F-1, FN-10
**Median bodyweight in kg (IQR)**	15.4 (8-27.6)
**Mean BCS/9 (*n* = 25) ± SD (95% CI)**	4.12 ± 1.56 (3.48-4.77)
**Breeds**	Cross (*n* = 5) and pure breeds (*n* = 22); Labrador Retriever (*n* = 3), Boxer (*n* = 2), Rottweiler (*n* = 2), and one each of German Shepherd dog, Cocker Spaniel, French Bulldog, Coonhound, Bichon Frisé, Pug, Kerry Blue, Tibetan Terrier, Fox Terrier, Chesapeake Bay Retriever, Lhasa Apso, German Shorthaired Pointer, West Highland White Terrier, Wheaten Terrier, and Border Terrier

Data in bold indicates demographic factors assessed in the neutrophilic inflammatory enteropathy group (*n* = 27). Abbreviations: BCS = body condition score; F = female; FN = female neutered; M = male; MN = male neutered.

The median duration of clinical signs before referral (available for 25/27 dogs) was 4 (3-10) weeks, with 4 (16%) cases having a duration of clinical signs of less than 3 weeks. Reported clinical signs before referral included small intestinal diarrhea (*n* = 21/27; 77.78%), vomiting (*n* = 21/27; 77.78%), weight loss (*n* = 20/27; 74.07%), hyporexia/anorexia (*n* = 13/27; 48.15%), and melena (*n* = 7/27; 25.93%). Twenty-five (92.59%) dogs had either diarrhea, vomiting, or a combination of these 2 clinical signs. The remaining 2 dogs presented with melena, hyporexia, and weight loss and melena alone, respectively. Treatment information for the 2 weeks before referral was available for all dogs and included antimicrobial therapy in 18 dogs, glucocorticoids in 9 dogs, a combination of antimicrobial therapy and glucocorticoids given concurrently in 6 dogs, and omeprazole in 9 dogs. Of the dogs that received antimicrobial therapy, metronidazole was the drug most frequently administered. Fifteen (55.56%) dogs had extra-gastrointestinal disease diagnosed, either before or at the time of diagnosing NIE (Appendix).

### Clinicopathological and diagnostic imaging findings

Hematology and serum biochemistry results were available for all 27 dogs. The median neutrophil count was 9600 (7850-16 600)/μL, with 12 (44%) dogs having values above the RI, while the median lymphocyte count was 1160 (530-1740)/μL, with 11 (41%) dogs having values below the RI. The mean albumin concentration was 25.28 (±6.07, 22.88-27.68) g/L, with 14 (52%) dogs having values below the RI, the mean globulin concentration was 21.97 (±5.42, 19.82-24.11) g/L, with 23 (85%) dogs having values below the RI, and the median cholesterol concentration was 133.02 (93.58-189.48) mg/dL, with 2 (7%) dogs having values above and 11 (41%) dogs having values below the RI. Trypsin-like immunoreactivity (TLI), folate, and cobalamin results were available for 19 (71%), 23 (85%), and 24 (89%) dogs, respectively. The median TLI was 12.9 (9.45-47) μg/L, with 5 (26%) dogs having values above and 1 (5%) dog having a value (4.3 μg/L) below the RI. The median folate concentration was 13.8 (4.7-24) μg/L, with 12 (52.17%) dogs having values above and 7 (30%) dogs having values below the RI. The median cobalamin concentration was 214.5 (150-560) ng/L, with 1 (4%) dog having a value above and 14 (58%) dogs having values below the RI. Additional clinicopathological results can be found in the Appendix. An adrenocorticotropic hormone (ACTH) stimulation test was performed in 14 (52%) dogs and excluded hypoadrenocorticism in all, with a pre-ACTH median cortisol of 2.83 (1.34-4.4) μg/dL, and a post-ACTH median cortisol of 12.8 (8.97-16.22) μg/dL. In addition, 1 (4%) dog had a basal cortisol of 4.93 μg/dL, also ruling out hypoadrenocorticism. Eleven dogs (40.74%) had hypocholesterolemia, of which 6 (55%) had hypoadrenocorticism excluded, either with an ACTH stimulation test (*n* = 5) or a basal cortisol concentration (*n* = 1). Five cases with hypocholesterolemia did not have hypoadrenocorticism excluded; however, the lack of supportive electrolyte abnormalities and the lack of bilaterally small adrenals on abdominal imaging made this an unlikely diagnosis.

Fecal parasitology results were available for 19 (71%) dogs, with *Toxocara canis*, *Giardia duodenalis*, and *Cryptosporidium* identified separately in 3 cases. Fecal bacteriological cultures were performed in 6 (22%) cases and were positive in 3 dogs yielding nonspecific mixed fecal commensals in 2 dogs and *Campylobacter* in 1 dog.

Abdominal ultrasonography was performed in all cases. The frequency and description of GI abnormalities detected are presented in Table 2. Extraintestinal ultrasonographic abnormalities were also recorded, with intra-abdominal lymph node abnormalities (abnormal echogenicity ± increased size) identified in 12 (44%) dogs, hepatic abnormalities (abnormal echogenicity ± presence of nodules) identified in 7 (26%) dogs, and pancreatic abnormalities (abnormal echogenicity ± increased size ± irregular margination) identified in 9 (33%) dogs. One case had ultrasonographic evidence of moderate pancreatitis (with a mild increase in Spec PL of 262 ug/L) and suspected ulceration in the stomach and the jejunoileum. This case was managed with omeprazole, sucralfate, and misoprostol. Repeat ultrasound 4 days later revealed similar findings. Upper GI endoscopy demonstrated several small gastric erosions. Histology of the stomach and duodenum revealed no and mild neutrophilic infiltrations, respectively. A second case had a non-obstructing jejunal/ileal foreign body, which passed naturally, confirmed by repeat ultrasound 24 h later. Seven (26%) cases had ascites, while 11 (41%) dogs had reactive peritoneal fat. Focal reactive peritoneal fat was identified in 5 cases; one case each adjacent to the pylorus (ultimately diagnosed with a gastric carcinoma), stomach, pancreas, multiple jejunal segments, and ileum (this case was diagnosed with a transient foreign body). The remaining 6 cases had multifocal (*n* = 1) and generalized (*n* = 5) reactive fat patterns.

**Table 2:** Gastrointestinal ultrasonographical imaging findings for all dogs with neutrophilic inflammatory enteropathy (*n* = 27), with pathology described in terms of the presence of a mass lesion, abnormal wall layering or increased wall thickness for the stomach, and all sections of the small intestine.

**Area**	**Abnormality present (*n*; %)**	**Mass lesion (*n*; %)**	**Abnormal wall layering (*n*; %)**	**Increased wall thickness (*n*; %)**
**Stomach**	7 (26)	1 (4)	3 (11)	6 (22)
**Duodenum**	9 (33)		8 (30)	3 (11)
**Jejunum**	13 (48)		13 (48)	4 (15)
**Ileum**	4 (15)	1 (4)	2 (7)	3 (11)

### Gastrointestinal biopsies and grading

All cases had endoscopy performed 1 day (*n* = 15), 2 days (*n* = 4), 3 days (*n* = 4), 5 days (*n* = 3), and 6 days (*n* = 1) after routine blood sampling. Gastrointestinal samples were acquired by either endoscopy (*n* = 26) or surgery (*n* = 2). Upper GI endoscopy was performed in 26 dogs and 7 of these also had lower GI endoscopies performed. No case had only lower GI endoscopy performed. One case had an upper GI endoscopy performed resulting in an inconclusive diagnosis for gastric neoplasia and subsequently surgical biopsies were performed; gastric neoplasia was then diagnosed. This case had gastric, jejunal, and ileal samples collected surgically, and histology regrading was performed on the full-thickness surgical biopsies. Overall, 26 gastric, 25 duodenal, 8 ileal, and 2 jejunal samples were examined. Biopsy samples were adequate for histopathological interpretation in all cases.

Each case had neutrophilic inflammation identified in either 1 (*n* = 18; 66%) or 2 (*n* = 9; 33%) GI locations. When present in 1 GI location, the duodenum was the most affected site (*n* = 14), followed by the stomach (*n* = 2) and ileum (*n* = 1). When identified in 2 areas, the stomach and duodenum (*n* = 6) followed by the duodenum and ileum (*n* = 3) were the most affected. One dog had jejunal biopsies alone. There were no cases in which neutrophilic inflammation was identified as the sole inflammatory infiltrate. Overall, the most common location for neutrophilic inflammation was the duodenum with 7/25 (28%), 12/25 (48%), and 4/25 (16%) dogs having mild, moderate, and severe neutrophilic inflammation. For the 2 non-neutrophilic duodenal cases, 1 dog had mild ileal neutrophilic inflammation, while the second dog had mild gastric neutrophilic inflammation. The most common concurrent inflammatory infiltrate in the duodenum was lymphoplasmacytic inflammation, which was mild, moderate, and severe in 2/25 (8%), 10/25 (40%), and 13/25 (52%) dogs, respectively. Within the ileum, 4/8 (50%) dogs had neutrophilic inflammation; mild in 3 (37.5%) dogs and severe in 1 (12.5%) dog. Of the 4 dogs that did not have neutrophilic ileal inflammation, 1 had severe gastric neutrophilic inflammation, 1 had severe duodenal and mild gastric neutrophilic inflammation, 1 had moderate duodenal neutrophilic inflammation, and the final dog had mild duodenal neutrophilic inflammation. The most common concurrent ileal inflammatory infiltrate was lymphoplasmacytic inflammation, which was mild, moderate, and severe in 2/8 (25%), 3/8 (38%), and 2/8 (25%) dogs, respectively. One (13%) dog had normal lymphoplasmacytic presence in the ileum. Gastric neutrophilic inflammation was identified in 8/26 (31%) dogs; mild, moderate, and severe in 5/26 (19%), 2/26 (8%), and 1/26 (4%) dogs, respectively. The remaining 18 (69%) dogs did not have gastric neutrophilic inflammation, but 17 (94%) of these had duodenal neutrophilic inflammation; mild, moderate, and severe in 5, 10, and 2 dogs, respectively, and the final dog had mild ileal neutrophilic inflammation. Two dogs that lacked gastric neutrophilic inflammation and had severe duodenal neutrophilic inflammation concurrently had mild (*n* = 1) and severe (*n* = 1) ileal neutrophilic inflammation, respectively. Two jejunal biopsies were assessed, in which neutrophilic inflammation was absent in one and severe in the second. The case in which it was absent had severe gastric neutrophilic inflammation. The most common concurrent jejunal inflammatory infiltrates identified in these 2 cases were moderate lymphoplasmacytic and moderate eosinophilic infiltrates.

### Fluorescent in situ hybridization (FISH)

Five (19%) cases, all belonging to the major NIE group, had FISH analysis performed, all with negative results. However, 3 of these cases received antimicrobial therapy and 1 dog received glucocorticoid therapy in the 2 weeks before referral.

### Treatment after diagnosis

Treatment after diagnosis is summarized in Table 3. Seven dogs were already receiving an appropriate diet and underwent no additional dietary change, resulting in dietary management for 24 (89%) dogs in total. The diets used included a hydrolyzed diet (*n* = 14), a low-fat diet (*n* = 7), and one each of a sensitivity diet, a novel protein diet, and a combination of sensitivity and hydrolyzed diets. No dog had diet alone as sole treatment after a diagnosis of NIE. Three dogs did not undergo dietary changes: in 1 case, a hydrolyzed diet was recommended, which the owner declined, another case did not undergo a diet change initially because of a poor appetite but was subsequently transitioned to a hydrolyzed diet almost 3 months later, to which the dog responded well; finally, one dog did not undergo a diet change for an unknown reason.

**Table 3:** Treatments used after diagnosis of NIE in the entire cohort (*n* = 27).

	**Treatments used after diagnosis of NIE**				
	**Dietary management**	**Glucocorticoid**	**Antimicrobial therapy**	**Glucocorticoids and antimicrobials**	**Additional immunosuppressants**
**Number of dogs (%)**	24 (89%)	18 (67%)	20 (74%)^a^	11 (41%)	3 (11%)

Abbreviation: NIE = neutrophilic inflammatory enteropathy.

a Four dogs received antimicrobial therapy for extra-gastrointestinal disease including one each of aspiration pneumonia, septic peritonitis post-exploratory laparotomy, bacterial cystitis, and skin disease.

Twenty (74.07%) dogs received antimicrobials, while 18 (66.67%) dogs received glucocorticoids, with 11 dogs receiving both. Of the 18 dogs treated with glucocorticoid therapy after referral, 17 received an immunosuppressive dose; 2 dogs started with an anti-inflammatory dose that was subsequently escalated to an immunosuppressive dose. Three cases had additional immunosuppressive agents administered, including ciclosporin (*n* = 2) and chlorambucil (*n* = 1). Other medications used included omeprazole (*n* = 16), cobalamin (*n* = 11), fenbendazole (*n* = 9), maropitant (*n* = 6), clopidogrel (*n* = 4), ondansetron (*n* = 3), metoclopramide (*n* = 3), domperidone (*n* = 2), and one case each for misoprostol, pro and prebiotic (Pro-Kolin), probiotic, and mirtazapine. The most commonly used antimicrobial therapies included metronidazole (*n* = 9), enrofloxacin (*n* = 9), amoxicillin-clavulanate (*n* = 5), cefalexin (*n* = 1), and azithromycin (*n* = 1), with 6 (30%) dogs receiving 2 different agents. One dog was prescribed azithromycin because of a *Cryptosporidium* infection of the GI tract. Four dogs had extra-GI disease necessitating antimicrobial use. One dog was treated with amoxicillin-clavulanate because of a clinical and radiographic suspicion of aspiration pneumonia. A second dog was diagnosed with a septic abdomen after exploratory surgery 5 days previously, receiving amoxicillin-clavulanate and enrofloxacin. A third dog received enrofloxacin for a bacterial cystitis. A fourth dog developed skin disease after endoscopy and received cefalexin.

### Overall grouping of neutrophilic inflammation: minor vs major neutrophilic groups alongside minor and major lymphoplasmacytic categorization

Overall, 8 (30%) cases had mild, 13 (48%) cases had moderate, and 6 (22%) cases had severe neutrophilic infiltration, resulting in a final categorization of minor and major neutrophilic inflammation in 8 (30%) and 19 (70%) dogs, respectively. Lymphoplasmacytic inflammation in the same cohort was mild in 2 (7%), moderate in 10 (37%), and severe in 15 (56%) dogs, resulting in a final categorization of minor and major lymphoplasmacytic inflammation in 2 (7%) and 25 (93%) dogs, respectively. Of the 8 minor NIE dogs, 1 had a concurrent minor lymphoplasmacytic inflammation, while the remaining 7 dogs had major lymphoplasmacytic inflammation. Of the 19 dogs in which major NIE was diagnosed, 1 dog had a concurrent minor lymphoplasmacytic inflammation, while the remaining 18 dogs had major lymphoplasmacytic inflammation. The median (IQR) duration of clinical signs before referral (available for 6 minor and 19 major NIE groups) was 3.5 (1.8-4) weeks for the minor NIE group and 6 (3.5-16) weeks for the major NIE group (*P* = .05). The mean (±SD) and median (IQR) hematological and biochemical variables, stratified between major and minor NIE groups, are detailed in Table 4. Cobalamin was the only variable that was significantly different between the 2 groups, with a lower concentration identified in the major NIE group. Overall median (IQR) hospitalization time was 4.0 (3-8) days. The hospitalization time for the major and minor NIE groups was not significantly different, at 4.0 (3-8) and 3.5 (2-11.8) days, respectively.

**Table 4:** Relevant clinicopathological data, stratified between major and minor neutrophilic inflammatory enteropathy groups, expressed as mean (±SD) or median (IQR). IQR expressed as 25th-75th percentiles.

**Variable**	**Major neutrophilic group; mean (**±**SD) or median (IQR)**	**Minor neutrophilic group; mean (**±**SD) or median (IQR)**	***P* value**
**HCT**	38 (±9)	40 (±14)	.62
**Neutrophil count**	9700 (7600-16 600)	9040 (8180-19 300)	.96
**Platelet count**	352 000 (295 000-544 000)	470 000 (314 000-568 250)	.63
**Lymphocyte count**	1160 (530-1860)	690 (520-1690)	.6
**Eosinophil count**	230 (30-510)	260 (70-615)	.61
**Albumin**	24.41 (±6.49)	27.35 (±4.61)	.26
**Globulin**	20.84 (±5.31)	24.64 (±5)	.1
**Cholesterol**	121.42 (84.69-158.93)	186.39 (129.54-221.58)	.06
**Total calcium**	8.9 (±1.12)	9.58 (±1.08)	.16
**Folate**	11.7 (4.6-24.1)	13.9 (8.23-16)	.97
**Cobalamin**	**188 (150-411.5)**	**489 (239-953)**	**.02** ^**a**^

Data in bold indicates variables assessed in dogs with neutrophilic inflammatory enteropathy (*n* = 27) and the variable with statistical significance between major and minor groups. Abbreviation: HCT = hematocrit.

a *P* < .05 considered statistically significant.

### Survival after discharge

**Figure 1:**
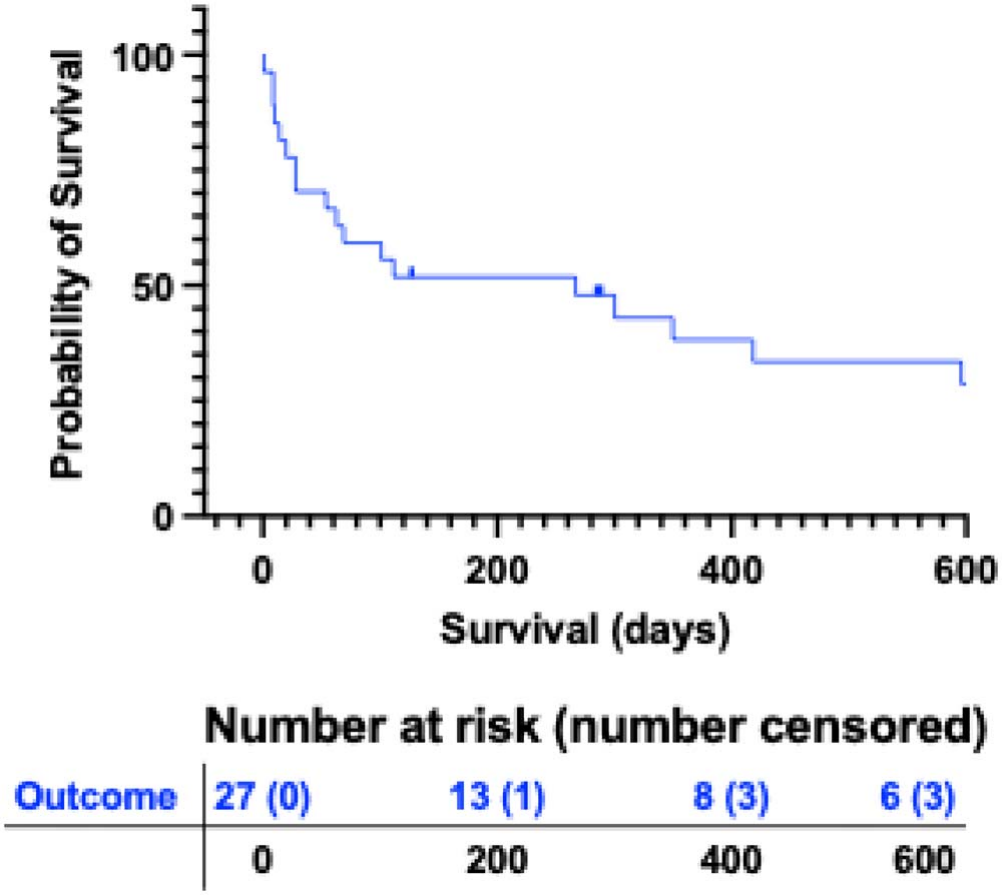
Kaplan–Meier survival curve for neutrophilic inflammatory enteropathy group (*n* = 27), with an overall median survival time of 267 days (95% CI, 0-569).

**Figure 2:**
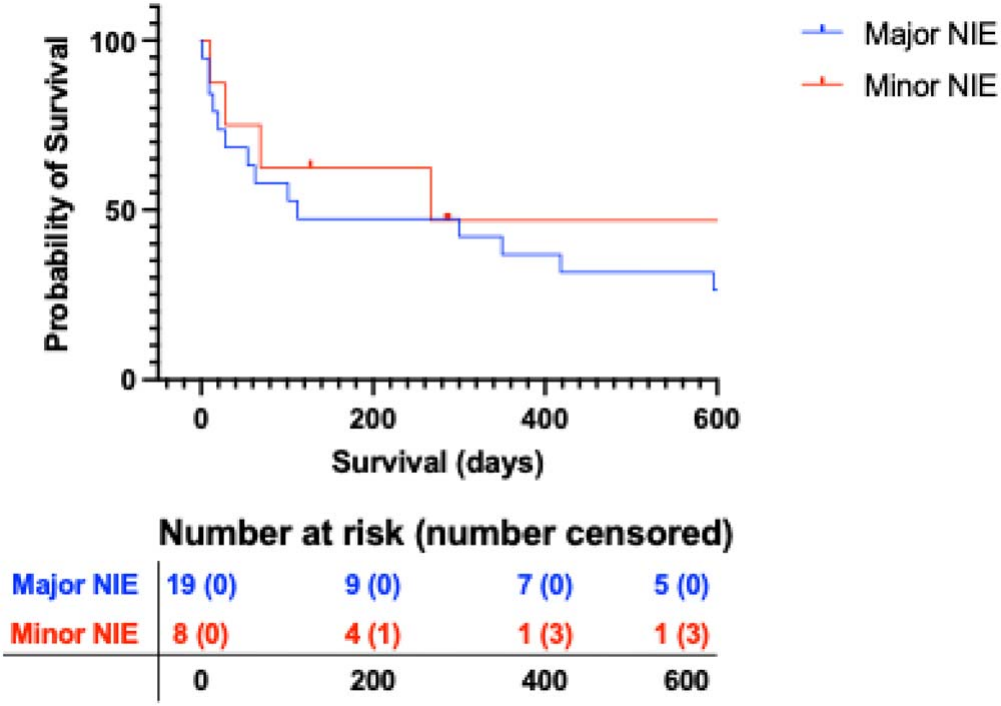
Kaplan–Meier survival curve for neutrophilic inflammatory enteropathy dogs (*n* = 27), stratified between major (blue line, *n* = 19) and minor (red line, *n* = 8) neutrophilic groups. MST for the major NIE group was 112 days and the MST for the minor NIE group was 267 days. No statistically significant difference was found between groups (*P* = .6). Abbreviations: MST = median survival time; NIE = neutrophilic inflammatory enteropathy.

Twenty-four (89%) dogs survived to discharge. Three cases did not survive, including 1 dog in the major and 2 dogs in the minor NIE groups. All 3 dogs were euthanized in the hospital because of their GI disease and worsening or ongoing hypoalbuminemia with the development of pleural effusion in one case and pitting edema in another, despite ongoing medical management. The overall median survival time (MST) for the NIE dogs was 267 (95% CI, 0-569) days (Figure 1), with no statistically significant difference between minor and major groups (Figure 2). At study end, 3 (11.11%) dogs were alive (survival times of 899, 694, and 288 days), 16 (59%) dogs had GI-related deaths, 4 (15%) dogs had GI-independent deaths (one each of liver disease, protein-losing nephropathy, acute kidney injury, and hemoabdomen), 3 (11%) dogs were lost to follow up, with 2 of these dogs having no signs of gastrointestinal disease reported at the time of last contact, and 1 (4%) died of an unknown cause but had persistent signs of gastrointestinal disease 1 month before death.

**Figure 3:**
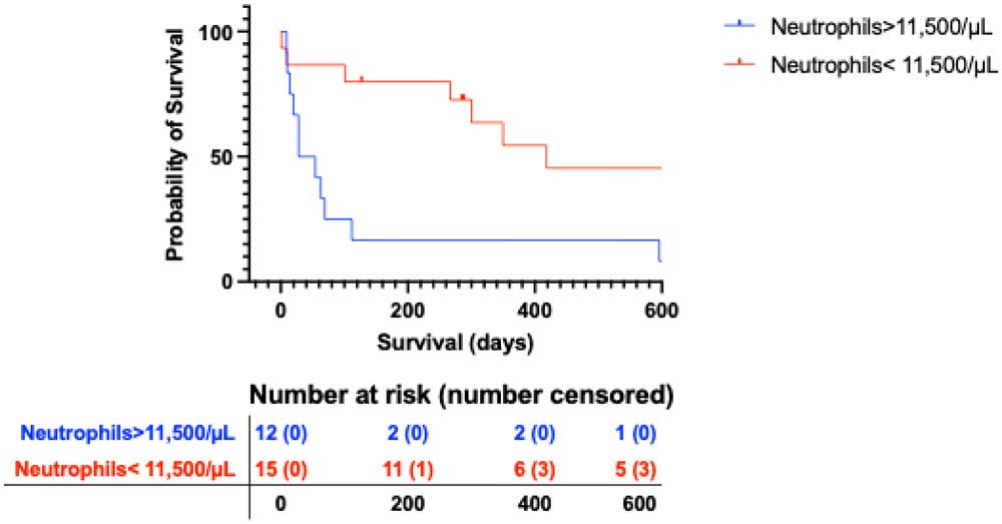
Kaplan–Meier survival curve for neutrophilic inflammatory enteropathy group (*n* = 27), stratified on the presence (blue line; *n* = 12) or absence (red line; *n* = 15) of peripheral neutrophilia (*n* = 27). An MST of 28 (95% CI, 0-66) days was found for those with peripheral neutrophilia, while an MST of 418 (95% CI, 0-1048) days was identified for those dogs without peripheral neutrophilia. A statistically significant difference was found using the log rank test (*P* = .007). Peripheral neutrophilia was defined as a peripheral neutrophil count of > 11 500/μL. Abbreviation: MST = median survival time.

Univariate Cox regression analysis demonstrated a hazard of 4.43 (95% CI, 1.16-16.99; *P* = .03) for the presence of peripheral neutrophilia, 3.16 (95% CI, 0.93-10.78; *P* = .07) for total hypocalcemia, 1.8 (95% CI, 0.57-5.58; *P* = .32) for hypocholesterolemia, 1.5 (95% CI, 0.43-5.23; *P* = .53) for hypocobalaminemia, and 1.48 (95% CI, 0.47-4.69; *P* = .5) for hypoalbuminemia. A multivariable Cox proportional hazard regression model was constructed using the peripheral neutrophil count and total calcium concentration. The overall model was statistically significant (*P* = .03); however, neither variable remained individually statistically significant, with peripheral neutrophilia having a hazard ratio of 3.59 (95% CI, 0.88-14.63; *P* = .07) and hypocalcemia having a hazard ratio of 2.2 (95% CI, 0.64-8.03; *P* = .23). Using Kaplan–Meier survival analysis, peripheral neutrophilia, which was identified in 12 (44.44%) dogs, was associated with an MST of 28 (95% CI, 0-66) days, whereas the MST for dogs without neutrophilia was 418 (95% CI, 0-1048) days (Figure 3). Comparison between these 2 groups was statistically significant (*P* = .007).

Univariate Cox regression analysis was performed to examine the effects of glucocorticoid and antimicrobial therapy after endoscopy. Neither glucocorticoid nor antimicrobial use after endoscopy was associated with a statistically significant increased hazard of death (3.12 [95% CI, 0.68-14.34; *P* = .14] and 1.06 [95% CI, 0.29-3.92; *P* = .93], respectively). As a change in diet was not the sole treatment in any dog, and because no consistent dietary recommendations were enforced, further statistical analysis was not performed.

## Discussion

This study specifically investigates NIE in dogs and highlights the potentially poor prognosis associated with the disease. Major lymphoplasmacytic inflammation was common to both the minor and major NIE groups suggesting it is the neutrophilic component that is associated with poorer survival; however, this is a preliminary finding and further research in this area is needed. Univariate analysis confirmed that peripheral neutrophilia was associated with an increased hazard of death; however, this association was not significant on multivariable analysis. Survival analysis also showed a significantly poorer survival in dogs with neutrophilia compared to those without neutrophilia. Results concerning peripheral neutrophil counts should be interpreted with caution, especially as treatments before referral and concurrent disease, which was present in 8 out of 12 dogs with neutrophilia, might have influenced results. Three dogs with documented ongoing or worsening hypoalbuminemia did not survive to discharge, further highlighting the negative effect that protein-losing gastrointestinal disease can have on outcome.

Neutrophils are the first immune cells to sites of inflammation, but if this process is not correctly regulated, their presence can cause tissue damage, predisposing to chronic disease.^18^ In both humans and animals, neutrophils are not present in the GI mucosa of healthy individuals.^15^^,^^18^ By contrast, they are present in humans with Crohn’s disease (CD) and ulcerative colitis (UC) and are recognized to play a crucial part in the overall inflammatory process. Their role in these 2 diseases differs considerably, typically being upregulated in UC and downregulated in CD. In UC, the extent of neutrophil infiltration contributes to the severity of the UC score assigned to an individual,^19^ signifying their importance. By contrast, there is a defect in neutrophil recruitment in CD. Inflammation involving neutrophils has been recognized to occur as part of the overall spectrum of canine CIE, however, very little is known about this finding. In this cohort of dogs, the presence of neutrophilic infiltration, alongside lymphoplasmacytic inflammation, was associated with a poor MST.

Peripheral neutrophilia is commonly identified in dogs and has many potential causes. Specifically, GI disease can result in neutrophilia either secondary to the underlying inflammatory disease, through exposure to bacterial products, or potentially through bacterial translocation and sepsis. It is clear from human studies that aberrant immunological responses in the GI tract can affect the epithelial barrier, thus increasing permeability to novel antigens.^18^ The prevalence of bacterial translocation in dogs, secondary to CIE, is largely unknown; however, a prospective study investigating the frequency of bacteremia secondary to acute hemorrhagic diarrhea syndrome (AHDS) suggests it occurs infrequently.^20^ Although AHDS is a different disease, it is associated with mucosal damage, and, in comparison to CIE, would likely overestimate the frequency of bacteremia in CIE. Of the 3 dogs that did not survive to discharge, 2 of which had neutrophilia, one was diagnosed with SIRS/sepsis, thought to be secondary to GI translocation. This case had concurrent pancreatitis, based on supportive abdominal ultrasound findings, which might also have contributed to death. Peripheral neutrophilia, in different disease states, is associated with a poorer prognosis, including in multicentric lymphoma^21^ and chronic hepatitis.^22^ The presence of peripheral neutrophilia in this study was also associated with a statistically significant increased hazard of death and poorer MST, suggesting it could be a promising biomarker for disease severity.

Dogs in this retrospective series presented with typical signs of gastrointestinal disease, associated with CIE, such as diarrhea, vomiting, and hyporexia/anorexia. However, the presence of melena in over a quarter of cases is notable. Melena might have been related to concurrent gastrointestinal disease, for example, one dog was diagnosed with gastric adenocarcinoma, one was diagnosed with possible gastrointestinal ulceration and a heterogeneous pancreas, raising concern for concurrent pancreatitis, and another dog was diagnosed with a transient foreign body. The remaining cases did not have an obvious cause identified. In human studies, neutrophils have been implicated in bleeding. One such study described increased numbers of macrophages and neutrophils in gastric ulcer margins of patients that rebleed.^23^ Another study demonstrated that neutrophils, through their interaction with the endothelium, were involved in thrombocytopenic bleeding.^24^ A further study identified significantly more intracranial bleeding and worse outcomes at 3 months in patients with higher neutrophil counts treated with recombinant tissue plasminogen activator for ischemic stroke.^25^ The increased frequency of GI bleeding in the current study could support the possibility that neutrophils are implicated in bleeding. Melena was identified in 3 minor, as well as 4 major, NIE cases, which might suggest that it is not the number of neutrophils that might predispose to melena, but rather their presence. This theory must be taken in context as neutrophilic infiltrates were not the only inflammatory infiltrates identified in dogs with melena, with concurrent major lymphoplasmacytic inflammatory infiltrates present in all dogs presenting with melena.

This study highlights important issues surrounding antimicrobial use in dogs with GI disease. Given the limited evidence to support antimicrobial treatment of CIE, the high prevalence is worrying. One likely explanation is that, despite limited evidence of an infectious etiology, an assumption was made that neutrophilic GI disease could have a similar etiology to (pyo)granulomatous large intestinal disease, with the latter attributed to adherent-invasive *Escherichia coli*^17^; however, a 0% prevalence of ARE was identified in a cohort of 60 dogs with CIE, calling into question its existence as a distinct disease entity.^12^ Antimicrobial therapy, with drugs such as metronidazole, negatively affects the GI microbiome, with 1 study showing a decrease in overall diversity along with a decrease in certain key bacteria, persisting for a minimum of 4 weeks after discontinuation of a 2-week course of the drug.^26^ Antimicrobial use is therefore not justified and should be avoided as it could have long-lasting adverse effects in CIE cases. Prescribing practices have changed at our institution in recent years; 7/12 (58.33%) dogs in this study received antimicrobial therapy after an NIE diagnosis between 2020 and 2025, compared to 13/15 (86.66%) dogs between 2015 and 2020. This trend presumably reflects a better understanding of the potential harmful effects of antimicrobial therapy in CIE cases. Fluorescent in situ hybridization analysis, performed in a small number of cases, was negative suggesting that, unlike (pyo)granulomatous disease of the large intestine, neutrophilic inflammation of the GI tract does not appear to have an infectious etiology; however, pretreatment with antimicrobials might have altered this interpretation.

### Limitations

This study had several limitations, not least its retrospective nature and incomplete clinicopathological data, including a lack of ACTH stimulation testing in 5 dogs. Another limitation is the relatively low number of cases which could have limited the analyses made and conclusions drawn. Survival analysis was based on the presence of neutrophilic inflammation, but this was not the only type of inflammatory infiltrate present in the biopsies, potentially confounding the results. The lack of consistent dietary changes after histological diagnosis before the addition of medications, such as glucocorticoids, might have contributed to the poor outcome seen in some dogs. Each case in which NIE was diagnosed histologically was presumed to have chronic enteropathy; however, it is possible that some dogs had acute disease. Given that the median duration of clinical signs before referral was 4 weeks, acute disease was considered infrequent. It is also possible that NIE was not primary in all cases; however, the retrospective nature of the study did not allow us to fully exclude all diseases that might have caused neutrophilic inflammation. Dogs frequently received treatment trials before referral and GI biopsy. This could have led to changes in hematological and biochemical variables as well as the histology in some cases, possibly reducing neutrophilic or lymphoplasmacytic components, potentially altering the classification of some neutrophilic cases and decreasing the severity of lymphoplasmacytic inflammation in others. The use of antibiotic therapy before referral might have also resulted in false negative FISH results and therefore these results should be interpreted with caution. Data were recorded for 2 weeks before referral as longer histories were often not available. Ideally, at least a 4-week history before referral would be required to determine whether longer courses of glucocorticoids or antimicrobial therapies were used, which could then influence results. Dogs with NIE were grouped into 2 categories which might be an oversimplification of the disease and could mask differences between mild and moderate and between moderate and severe neutrophilic cases. However, at our institution (and elsewhere) it is common for histology reports to define inflammatory GI disease overall as either mild or moderate/severe; therefore, reporting in this way reflects clinical practice in many institutes.

## Conclusions

Dogs with neutrophilic gastrointestinal disease had a poor outcome. Peripheral neutrophilia might act as a prognostic marker to help predict disease severity and outcome; but caution is required in peripheral neutrophil data interpretation because of previous treatments and concurrent disease.

## Off-label antimicrobial declaration

The authors declare no off-label use of antimicrobials.

## Institutional animal care and use committee or other approval declaration

The authors declare no institutional animal care and use committee or other approval was needed.

## Appendix

### Neutrophilic inflammatory enteropathy grading

Neutrophilic gastric and small intestinal (duodenal and ileal) inflammation was graded as follows (cells per 400× field): **absent**, if no neutrophils were present in the lamina propria of the stomach or small intestine; **mild**, if ≤ 20 neutrophils were present in the lamina propria of the stomach or ≤ 10 neutrophils in the lamina propria of the small intestine; **moderate**, if 21-50 neutrophils were present in the lamina propria of the stomach or 11-30 neutrophils in the lamina propria of the small intestine, and **severe**, if ≥ 51 neutrophils were present in the lamina propria of the stomach or ≥ 31 neutrophils in the lamina propria of the small intestine, as per Allenspach et al. (2019). Grades were separately assigned to the gastric and small intestinal sections available for each case. An overall severity score of mild, moderate, or severe neutrophilic inflammation was assigned to each case, based on the highest score from all available tissues. Each case was then allocated to a group of minor neutrophilic inflammation, if the overall severity score was mild, or major neutrophilic inflammation, if the overall severity score was moderate or severe.

### Lymphoplasmacytic inflammatory enteropathy grading

Lymphoplasmacytic gastrointestinal inflammation was graded as follows: **Gastric** (cells per 400× field): **Normal** ≤ 20, **Mild** 21-50, **Moderate** 51-100, and **Severe** ≥ 101, cells per 400× field and **Small intestine** (% area of one 400× villous field or cells between crypts): **Normal** ≤ 25, ≤ 2, **Mild** 26-50, 3-5, **Moderate** 51-75, 6-10, and **Severe** ≥ 76, ≥11), as per Allenspach et al. (2019). Grades were separately assigned to the gastric and small intestinal sections available for each case. An overall severity score of mild, moderate, or severe lymphoplasmacytic inflammation was assigned to each case, based on the highest score from all available tissues. Each case was then allocated to a group of minor lymphoplasmacytic inflammation, if the overall severity score was mild, or major lymphoplasmacytic inflammation, if the overall severity score was moderate or severe.

### Diarrhea definition

Diarrhea was defined as small intestinal in origin by the presence of increased frequency and fluid consistency of feces, without tenesmus or mucus. Additional clinical signs recorded included weight loss, hyporexia/anorexia, melena, and vomiting.

### Hematology, serum biochemistry, TLI, folate, cobalamin, and cortisol analyzers and reference intervals used

Hematology and serum biochemistry were performed using the same analyzers (Advia 2120 and Atellica, respectively; Siemens Healthineers). Reference intervals for relevant clinicopathological data included hematocrit (HCT): 37%-55%, reticulocyte count: 0-60 000/μL, mean corpuscular volume (MCV): 60-77 μm^3^, platelet count: 150 000-500 000/μL, neutrophil count: 3000-11 500/μL, lymphocyte count: 1000-3600/μL, eosinophil count: 0-1470/μL, albumin: 25-38 g/L, globulin: 28-42 g/L, cholesterol: 123.74-251.35 mg/dL, total calcium: 9.22-12.02 mg/dL, and 1,2-o-dilauryl-rac-glycero-3-glutaric acid-(6′-methylresorufin) ester (DGGR) lipase: 0-130 U/L. Ionized calcium was recorded where available and assessed using the RAPIDPoint 500 (Siemens Healthineers) analyzer, using a reference interval (RI) of 4.49-5.61 mg/dL. Trypsin-like immunoreactivity (TLI), folate, and cobalamin, measured using the Immulite 2000 platform (cTLI, FOL, VB, respectively; Siemens Healthineers), were recorded where available, using RIs of 5-45 μg/L, 8.2-13.5 μg/L, and > 275-1000 ng/L, respectively. Cortisol concentrations (Canine Cortisol, Immulite 2000; Siemens Healthineers), before and after ACTH stimulation testing, were recorded where available, using RIs of 1.81-5.44 and 5.44-16.31 μg/dL, respectively.

### Abdominal ultrasonography

Abdominal ultrasonography was performed using the LOGIQ E9 ultrasound machine (model number; VA-26834-2), by 1 of 4 board-certified diagnostic imaging European specialists, or a diagnostic imaging resident under their supervision.

### Treatments and dietary history before and after referral

Treatments for 2 weeks before referral and all treatments after referral were recorded. Diet history before referral was not routinely described but, where available, was recorded. The feeding of a raw diet is routinely investigated at our institution and recorded in referral letters; no cases in this cohort had a history of raw feeding.

### Outcome measures

Outcome measures, including hospitalization time and survival after discharge, were recorded.

### Extra-gastrointestinal diseases diagnosed before or at the time of neutrophilic inflammatory enteropathy diagnosis

Fifteen (55.56%) dogs had one or more diseases diagnosed either before or at the time of diagnosing neutrophilic inflammatory enteropathy, including myxomatous mitral valve disease (*n* = 3), pancreatitis (*n* = 3), gastric adenocarcinoma and hepatitis (*n* = 1), aspiration pneumonia (*n* = 1), mammary mass of unknown type (*n* = 1), unilateral ureteral obstruction and hydronephrosis (*n* = 1), chronic untreated seizure activity (*n* = 1), nephrolithiasis, cystolithiasis, and *Escherichia coli* urinary tract infection (*n* = 1), intervertebral disc disease requiring hemilaminectomy (*n* = 1), chronic kidney disease/protein losing nephropathy and possible sub-aortic stenosis (*n* = 1); immature gallbladder mucocele, primary hypothyroidism, and unilateral renal agenesis (*n* = 1), leishmaniasis (under treatment with allopurinol, serologically under control), nephrolithiasis and cystolithiasis (*n* = 1), septic peritonitis after exploratory laparotomy (*n* = 1), and incidentally discovered hepatic cysts (*n* = 1). The dog diagnosed with hepatitis had a surgical biopsy performed at the time of intestinal biopsies and a mild/moderate chronic active histiocytic, lymphoplasmacytic, and neutrophilic periportal hepatitis with nodular hyperplasia was diagnosed. One dog had a diagnosis of pancreatitis based on ultrasound findings; however, no cranial abdominal pain was reported, and the dog had a normal 1,2-o-dilauryl-rac-glycero-3-glutaric acid-(6′-methylresorufin) ester (DGGR) lipase activity. A second pancreatitis diagnosis was based solely on clinical history and was not active at the time of referral and the final diagnosis of pancreatitis was based on increased Spec PL (PLI) of 262 μg/L (normal < 200 ug/L) and abnormal pancreatic ultrasonography.

### Additional clinicopathological data available for all 27 neutrophilic inflammatory enteropathy dogs

The mean HCT was 39 (±10, 35-43)%, with one (3.7%) dog having a value above and 11 (40.74%) dogs having values below the RI, the median reticulocyte count was 51 000 (38 000-88 400)/μL with 12 (44.44%) dogs having values above the RI, the median mean corpuscular volume (MCV) was 69.5 (67-70.4) μm^3^, with 1 (3.7%) dog having a value above and 2 (7.41%) dogs having values below the RI, the median platelet count was 412 000/μL (295 000-545 000) with 9 (33.33%) dogs having values above the RI and the median eosinophil count was 230 (40-510)/μL, with 3 (11.11%) dogs having values above the RI. The mean total calcium concentration was 9.1 (±1.16, 8.66-9.58) mg/dL, with 11 (40.74%) dogs having values below the RI and the median 1,2-o-dilauryl-rac-glycero-3-glutaric acid-(6′-methylresorufin) ester (DGGR) lipase was 42 (25-76) U/L, with 2 (7.41%) dogs having values above the RI. Ionized calcium was available for 13 dogs and a mean ionized calcium of 4.45 (±0.44, 4.37-4.89) mg/dL was found, with 4 (30.77%) dogs having values below the RI.

## Abbreviations

ACTH: adrenocorticotropic hormone
AHDS: acute hemorrhagic diarrhea syndrome
ARE: antibiotic responsive enteropathy
CD: Crohn’s disease
CIE: chronic inflammatory enteropathy
DGGR: 1,2-o-dilauryl-rac-glycero-3-glutaric acid-(6′-methylresorufin) ester
FISH: fluorescent in situ hybridization
GI: gastrointestinal
MCV: mean corpuscular volume
MST: median survival time
NIE: neutrophilic inflammatory enteropathy
PLI: pancreatic lipase immunoreactivity
RI: reference interval
SIRS: systemic inflammatory response syndrome
SPSS: statistical package for the social sciences
TLI: trypsin-like immunoreactivity
UC: ulcerative colitis
